# Chronic Diseases and Labor Force Participation Among Presenile and Senile Chinese

**DOI:** 10.3389/fpubh.2021.675927

**Published:** 2021-09-16

**Authors:** Xiaotuo Qiao, Bo Wang, Haifeng Guo

**Affiliations:** ^1^Finance, School of Management, Harbin Institute of Technology, Harbin, China; ^2^Economics, School of Humanities, Social Science and Law, Harbin Institute of Technology, Harbin, China

**Keywords:** chronic diseases, labor force participation, mental problem, multivariate probit model, presenile and senile chinese

## Abstract

**Background:** The incidence of chronic diseases has increased dramatically due to rapid aging and lifestyle changes of China in recent decades. The population aged more than 45 years is an important participant in the labor force market, and the health status directly affects their labor force participation decision. This study aims to explore the relationship between chronic diseases and the labor force participation among the elderly Chinese population aged more than 45 years.

**Method:** We employ a multivariate probit (MVP) model to construct five structural equations for an analysis. The advantage of this model is that it can deal with the endogeneity of chronic diseases.

**Results:** Firstly, compared with the elderly, younger people are more likely to participate in the labor force market; the influence of chronic diseases is the largest for presenile women in the decision-making of labor force participation; the impact of psychological problems on labor force participation cannot be ignored, especially for men aged more than 45 years. In addition, sociodemographic factors such as geographical location and marital status also have direct effects on the probability of labor force participation while the impact of both family wealth and family number is much smaller. Finally, unhealthy lifestyles through chronic diseases have negative and indirect marginal effects on labor force participation.

**Conclusions:** This article proves that chronic diseases have a negative impact on the labor force participation for Chinese aged more than 45 years. The public should give more tolerance and opportunities to these groups. The population aged more than 45 years are more vulnerable and face more psychological problems, which will lead to a decline in labor force participation. Psychological health counseling and services are urgently needed. As the urban areas enjoy more social welfare, Chinese welfare policy needs to be tilted toward the rural elderly. For individuals, maintaining healthy lifestyles can help you stay away from chronic diseases and stay in the labor force market.

## Introduction

The global aging problem has become increasingly prominent, and the growth rate of the aging population of China ranks first in the world. In 2020, the population aged more than 40 years accounted for ~17.8% of the total population in China. It is expected that a quarter of the population will be more than 60 years old by 2,035 (WHO). The aging of population has led to the changes in disease patterns dominated by chronic diseases, which put tremendous pressure on the society and the economy (1). Chronic diseases have been described as a non-negligible problem accompanying the aging society. According to the Global Cancer Research Center, the number of chronic diseases in the global elderly population will boost from 12.7 million in 2008 to 21.5 million in 2030 (2). The epidemic of chronic diseases will provoke long-term and huge problems such as labor shortage, increasing dependency ratio, and crisis of social security system.

The relaxation of the one-child policy indicates that the demographic structure of China is unbalanced and skewing toward the elderly. Meanwhile, the prevalence of chronic diseases in the presenile and senile population poses a huge health challenge to the health targets of Millennium Development Goals. Under the current national conditions of China, presenile and senile workers occupy a vital position in the labor force market. However, the reality is that the labor force participation of these groups is quite low. The key reason lies on the ill-health and subhealth conditions caused by chronic diseases.

Chronic diseases will lead to subhealthy status, thereby affecting the labor force participation of an individuals. The relationship between chronic health conditions and the work status stems from the theoretical concepts of human resources and health production model, and health can be treated as an endowment of human capital (3). Poor health has a negative impact on labor force participation. Bloom et al. (4) summarized how health affects the economy: people with good health take a longer time to work and are more productive, healthy people have a longer life expectancy, so they have more motivation to invest in human capital that will also provide more investment to the economy. However, it is difficult for the elderly with chronic diseases to continue a remunerated work, and they tend to exit the labor force market earlier. On the other hand, when older people have no sources of income, they need the support from the labor population that will become a huge burden for social welfare (5).

However, there is no systematic research on the relationship between the health status and the labor force participation in China. Current literature studies mainly focus on developed labor markets such as Australia with adequate welfare and pension systems while there is a general lack of research on developing countries especially China, which is facing severe aging problem. In addition, traditional empirical studies of health status often use self-assessed health indicators, which will lead to an unobservable deviation and inaccuracy (6). Another problem is that current literature studies on health human capital focus on physical health rather than psychological health (7). There are no profound studies about the mental health impact on labor force participation (8, 9). Nevertheless, in a stressful contemporary society, mental problems such as presenile depression are emerging constantly. Measuring the impact of mental problems is obligatory (10). In this article, psychological problems are included in the research scope as a kind of chronic disease, which can enrich the existing human capital theory to a certain extent. Furthermore, it is necessary to analyze the joint pathology of chronic diseases. Most scholars only pay attention to the impact of certain health conditions or specific chronic diseases on labor force participation (11, 12). A few studies on the impact of multiple chronic diseases and comorbidity are limited. However, different chronic diseases may interact with each other, they will have a direct impact on labor force participation and an indirect impact through other chronic diseases (13). For example, as a diabetic complication, the prevalence of cardiovascular diseases in patients with diabetes is very high, about 72.2% in our data. As a consequence, we select the four representative chronic diseases with a high incidence among presenile and senile Chinese residents as our research objects, and estimate their unique and mutual influence on labor force participation using the multivariate probit (MVP) model (14). The four kinds of chronic diseases include cardiovascular diseases, diabetes, mental problems, and other chronic diseases such as cancer, asthma, and digestive diseases. The exogenous MVP model is a generalization of the probit model and is used to jointly estimate multiple correlated binary outcomes (15).

This article contains four main sections. section Materials and Methods introduces the method—MVP model, section Results presents the model results including various probabilities, treatment effects, and marginal effects, and section Discussion and Conclusion.

## Materials and Methods

### MVP Model

Chronic diseases possessing the characteristics of comorbidity, different chronic diseases may have similar influencing factors, and a separate analysis of each chronic disease cannot reflect the real impact on labor force participation. To solve this problem, our article chooses the MVP model to evaluate the impact of the four chronic diseases on the labor force participation of presenile and senile Chinese residents. The MVP model is suitable for analyzing both observable and unobservable effects on the labor force participation and the morbidity of chronic diseases. This model can identify the key factors affecting labor force participation and presume chronic diseases as endogenous (16). Most studies in this field suggest that chronic diseases are endogenous, but scholars do not have a perfect method to measure endogeneity, our model builds five simultaneous equations and chooses lifestyles and personal characteristics to control the endogeneity of chronic diseases. The MVP model is based on the multivariate normal distribution and is recommended when the relevant alternatives are independent of each other. This model is suitable for studying the impact of chronic diseases, which are closely related and tend to be affected by the same factors (17).

The impact of chronic diseases on labor force participation is indirectly replicated through human capital, where health is regarded as a form of human capital. The mechanism is that non-communication diseases affect the health of an individual, and the health status affects the labor supply. If the expected utility is >0, the individual chooses to participate in the labor market (18). We define a binary variable to model the outcome: *L* = 1 in case of participation in the labor force, and 0 in case of no participation. In addition, we set Ch = 1 if an individual suffers from a certain kind of chronic disease *h* and 0 if not. Individual choice is affected by the two ways: the observable factors and the unobservable factors of personal preference (19). The choice function can be described as follows:


(1) $$\begin{array}{l} \begin{array}{c} Y=\left\{\begin{array}{ccccc} 1 & \text{if} & {\text{Y}}^{*} & >\; & 0\\ 0 & \text{if} & {\text{Y}}^{*} & \leq & 0 \end{array}\right\} \end{array} \end{array}$$

The multiple-choice probability of an individual *i* in the MVP model can be described as:


(2) $$\begin{array}{l} \mathrm{Pr}ob(y_{i1}=1,y_{i2}=1,y_{i3}=1,y_{i4}=1,y_{i5}=1|x_{ij},\rho )\\ \;=\mathrm{Pr}ob(y_{i1}^{*}>0,y_{i2}^{*}>0,y_{i3}^{*}>0,y_{i4}^{*}>0,y_{i5}^{*}>0|x_{ij},\rho )\; \end{array}$$

The discrete stochastic model is presented as the following equations


(3) $$\begin{array}{l} \begin{array}{l} \begin{array}{l} L^{*}={\text{x}}_{L}^{\prime }\beta_{L}+\gamma_{H}c_{H}+\gamma_{D}c_{D}+\gamma_{M}c_{M}+\gamma_{O}c_{O}+\varepsilon_{L}\\ c_{H}^{*}={\text{x}}_{H}^{\prime }\beta_{H}+\varepsilon_{H}\\ c_{D}^{*}={\text{x}}_{D}^{\prime }\beta_{D}+\varepsilon_{D}\\ c_{M}^{*}={\text{x}}_{M}^{\prime }\beta_{M}+\varepsilon_{M}\\ c_{O}^{*}={\text{x}}_{O}^{\prime }\beta_{O}+\varepsilon_{O} \end{array} \end{array} \end{array}$$

where the stochastic error terms ε_*i*_ follow the multivariate normal distribution with mean 0 and variance Σ, that is (ε_*L*_, ε_*H*_, ε_*D*_, ε_*M*_, ε_*O*_)~ MVN[0, Σ].

This article studies the impact of chronic diseases on the decision-making of presenile and senile Chinese about whether to participate in the labor force market. The MVP model in this article contains five seemingly unrelated equations. The multivariate normal cdf and conditional probabilities can be described as Equations (4) and (5), respectively.


(4) $$\begin{array}{l} \begin{array}{c} \begin{array}{c} \mathrm{Pr}\;ob\left(X_{1}<x_{1},X_{2}<x_{2},X_{3}<x_{3},X_{4}<x_{4},X_{5}<x_{5}\right)=\int_{-\infty }^{x_{5}}\int_{-\infty }^{x_{4}}\int_{-\infty }^{x_{3}}\int_{-\infty }^{x_{2}}\int_{-\infty }^{x_{1}}\phi_{5}\left(Z_{5},Z_{4},Z_{3},Z_{2},Z_{1},\rho \right)dz_{1}dz_{2}dz_{3}dz_{4}dz_{5}\\ \;=\mathrm{Pr}\text{ob}\left(X_{1}=1,X_{2}=h,X_{3}=d,X_{4}=m,X_{5}=0\right) \end{array} \end{array} \end{array}$$

(5) $$\begin{array}{l} \begin{array}{c} \mathrm{Pr}\;ob\left(X_{1}=1|X_{2}=h,X_{3}=d,X_{4}=m,X_{5}=0\right)=\frac{\mathrm{Pr}\text{ob}\left(X_{1}=1,X_{2}=h,X_{3}=d,X_{4}=m,X_{5}=0\right)}{\mathrm{Pr}\text{ob}\left(X_{2}=h,X_{3}=d,X_{4}=m,X_{5}=0\right)} \end{array} \end{array}$$

The joint probabilities of the observed events [*L, C*_*H*_, *C*_*D*_, *C*_*M*_, *C*_*O*_] that form the basis of the log-likelihood function are the five-variate normal probabilities, and the log-likelihood equation is defined as:


(6) $$\begin{array}{l} \begin{array}{l} \;L_{i}=\Phi_{5}\left(q_{iL}{x^{\prime }}_{iL}\beta_{L},q_{iH}{x^{\prime }}_{iH}\beta_{H},q_{iD}{x^{\prime }}_{iD}\beta_{D},q_{iM}{x^{\prime }}_{iM}\beta_{M},q_{i0}{x^{\prime }}_{i0}\beta_{0},R^{*}\right)\\ \text{ where}\\ \;q_{im}=2y_{im}-1\\ \;R_{jm}^{*}=q_{ij}q_{im}\rho_{jm}\\ \;\text{m}\in \left(\text{L,H,D,M,O}\right)\\ \;{\text{q}}_{ij}=1\text{ if}{\text{y}}_{ij=1}\text{and}-\text{1 if }{\text{y}}_{ij}=0\text{ for j}=1,2,3,4,5 \end{array} \end{array}$$

In addition, we also calculate the treatment effects of having certain chronic diseases on labor force participation, it can be described as the difference between the conditional probabilities of labor force participation with and without that kind of chronic disease.

### Data and Sample

Our sample comes from the China Health and Retirement Longitudinal Study (CHARLS 2015) funded by the World Bank, Peking University, and the National Institute of Aging in China. The study uses a longitudinal questionnaire and collected the individual and household information of representative samples of Chinese residents aged more than 45 years. Recent CHARLS data were conducted in 28 provinces, 150 districts, and 450 villages across the country. It is the most reliable national source of information on aging, health conditions, and work status (4). For the purpose of comparison, the incidence of chronic diseases of residents aged more than 60 years is 55.1% in our sample, it is very close to the estimation of 51.2% in the Chinese Health Statistics Yearbook (CHSY).

We select the three waves from the CHARLS data set to conduct our analysis: (1) demographic background wave, (2) health status and functioning wave, and (3) healthcare and insurance wave. The sample in this article involves over 11,300 Chinese individuals aged more than 45 years (20). In this article, we define “presenile” as people aged more than 45 years and before retirement, “senile” as those who have retired. Based on this, we further define women between 45 and 55 years as the “presenile women” group, women more than 55 years as the “senile-women” group, and men between 45 and 60 years as the “presenile men” group, and men more than 60 years as the “senile men” group. After data cleansing, our sample includes the four groups of cross-sectional data.

The binary variable of the labor force participation derives from the “working status” of respondents. Our clarification of chronic diseases is based on the two questions of the CHARLS questionnaire—“Have you been diagnosed with the following chronic disease by a doctor (DA007)” and “Do you know if you have certain kind of chronic disease (DA008).” There are

14 kinds of chronic diseases included in the questionnaire. We define cardiovascular diseases as “hypertension; dyslipidemia; heart attack, coronary heart disease, angina, congestive heart failure, or other heart problems; stroke”; diabetes as “diabetes or high blood sugar”; mental problems as “emotional, nervous, or psychiatric problems”; other diseases include “cancer or malignant tumor; chronic lung diseases; liver disease; kidney; stomach; or other digestive disease; memory-related disease; and arthritis or rheumatism; Asthma.” The answers to these two questions only include “yes or no,” thus we can only know whether the respondent suffers from certain chronic disease or not. It is difficult to classify the level of each chronic disease and is impossible for us to use a dummy variable to define the different levels of chronic diseases in our analysis. All the definition of the abbreviations used in the model and later empirical analysis are listed in the Appendix at the end of the paper.

## Results

### Probabilities and Correlations

Before estimation, we first present a brief description of the variables used in the analysis. As shown in panel A of Table 1, the mean of labor force participation is 0.67 with a positive skewness. Most of the respondents are more than 45 years old with an average age of around 60 years; the sample is balanced in terms of gender and region, with little more women than men, and little more rural respondents than the urban ones. Most of the respondents are married, the mean is 0.87 with a negative skewness. The average household total wealth is 0.11 million, and the average number of each household is 3.4. In the sample, only a minority of respondents are highly educated, the mean of middle- and low-level education is 0.31 and 0.11, respectively, indicating that more than half of the sample respondents have no educational background. Regarding lifestyle variables, the mean of moderate drinking (MIDDRK) is 0.11, and the mean of heavy drinking (HVYDRK) is much lower, indicating that most of the respondents have no drinking habits; the same is true for smoking habits, the mean of current smoking (CURSMK), and historical smoking (BEFSMK) is 0.28 and 0.12, respectively, more than half of the respondents do not smoke. In terms of exercise, about 47% of the sample have high-intensity exercise, 37% have moderate-level exercise, and 7% have low-intensity exercise, and only a small number of respondents do not take exercise.

**Table 1:** Summary description.

**Variable**	**Mean**	**Median**	**std**	**Skewness**	**Kurtosis**
**A: DESCRIPTION OF CHARACTERISTICS**					
Labor	0.67	0	0.47	0.74	1.55
MALE	0.48	0	0.50	0.08	1.01
AGE	59.77	59	10.20	0.57	2.91
URBAN	0.41	0	0.49	0.39	1.15
MARRIED	0.87	1	0.33	−2.23	5.97
TOTWTH	11.31	11.47	1.44	−0.67	4.62
NUMBER	3.40	3	1.74	1.25	5.79
EDUHIG	0.03	0	0.17	5.55	31.85
EDUMID	0.31	0	0.46	0.85	1.72
MIDDRK	0.11	0	0.31	2.54	7.43
HVYDRK	0.07	0	0.26	3.36	12.28
CURSMK	0.28	0	0.45	0.99	1.99
BEFSMK	0.12	0	0.32	2.37	6.62
EXH	0.47	0	0.50	0.11	1.01
EXM	0.37	0	0.48	0.53	1.29
EXL	0.07	0	0.26	3.38	12.42
	**Labor**	**Card**	**Diab**	**Mental**	**Othdis**
**B: CORRELATION**					
Labor	1				
Card	−0.206^***^	1			
Diab	−0.108^***^	0.198^***^	1		
Mental	−0.045^***^	0.032^***^	0.013^*^	1	
Othdis	−0.017^**^	0.105^***^	0.022^***^	0.057^***^	1

* *means significant at the 10% level;*

** *means significant at the 5% level;*

*** *means significant at the 1% level*.

In panel B of Table 1, we give the simple pairwise correlations among labor force participation and the four kinds of chronic diseases. All four chronic diseases have significant negative correlations with labor force participation, the coefficients of two physical diseases are −0.206 and −0.108, and the coefficient of mental problems is smaller but unneglectable. The correlations of various chronic diseases are also significant among which the coefficient of cardiovascular diseases and diabetes is the highest, that is, 0.198, it indicates that a univariate analysis of each chronic disease is inaccurate and the consideration of comorbidity is necessary.

We also give a detailed analysis of the unconditional and some conditional probabilities of each chronic condition, which reflects the intangible heterogeneity of the observations in Table 2. From this table, we can see that the conditioning on other kinds of chronic diseases and the probability of certain chronic disease increase significantly. For instance, the unconditional probability of cardiovascular diseases (Ch) is 34.5% while the probability increases to 72.8% conditioning on diabetes. The conditional probabilities of diabetes (Cd) range from 6.2 to 16.9%, all higher than the unconditional probability of 5.7%. The same is true for the probability of mental problems and other chronic diseases. Significant correlations and high conditional probabilities of the four chronic diseases indicate that it is necessary to establish simultaneous equations and estimate the cross-equation error terms. Considering the high comorbidity, we choose a system function to calculate the correlated errors, which make it possible to estimate an unobservable impact after controlling the observable factors (21).

**Table 2:** Probability of chronic diseases.

	**C_**h**_**	**C_**d**_**	**C_**m**_**	**C_**o**_**
P(.)	34.5	5.7	2	53.1
P(.|*C_*h*_*=1)	100	12	2.7	60.2
P(.|*C_*d*_*=1)	72.8	100	2.8	57.4
P(.|*C_*m*_*=1)	45.3	7.9	100	72.7
P(.|*C_*o*_*=1)	39.3	6.2	2.8	100
P(.|*C_*h*_*=1, *C_*d*_*=1)	100	100	3.7	59.5
P(.|*C_*h*_*=1, *C_*m*_*=1)	100	16.9	100	84.6
P(.|*C_*d*_*=1, *C_*m*_*=1)	96.4	100	100	85.7

Our concern is to analyze the impact of chronic diseases on the labor force participation of Chinese workers in the context of aging, thus we split our sample into four groups—presenile men (age ≤ 60), senile men (age > 60), presenile women (age ≤ 55), and senile women (age > 55). In Table 3, we list the percentages of labor force participation under different chronic conditions for the four age-gender groups. Consistent with common sense, when one gets any chronic disease, the labor force participation rate will drop. For example, the labor force participation of men decreases from 80.33 to 61.03% when getting cardiovascular diseases. In the similar age groups, the labor force participation rate of men is higher than that of women in all age and chronic cases, taking the labor force participation with diabetes as an example, the rate for presenile men is 76.21%, higher than 60.45% of presenile women, the rate for senile men is 36.32%, also higher than 33.09% of senile women. On the other side, in the same gender groups, the labor force participation rate of preseniles is much higher than that of seniles regardless of chronic conditions. Such as the percentage for presenile men with diabetes, the value of 76.21% is more than two times of 36.32%—the percentage for senile men with diabetes.

**Table 3:** The labor force participation of each chronic disease (%).

	**Ch = 1**	**Ch = 0**	**Cd = 1**	**Cd = 0**	**Cm = 1**	**Cm = 0**	**Co = 1**	**Co = 0**
Male	61.03	80.33	55.00	75.10	54.29	74.42	73.55	74.73
Presenile Male (60–)	81.55	90.14	76.21	88.37	57.97	88.27	87.62	88.04
Senile Male (60+)	45.17	65.14	36.32	58.36	50.70	57.13	59.07	54.46
Female	47.88	67.93	39.82	61.88	50.71	60.70	59.99	61.02
Presenile Female (55–)	68.63	79.75	60.45	77.51	64.18	76.92	76.54	76.92
Senile Female (55+)	40.07	58.59	33.09	51.95	44.44	50.72	51.66	48.90

### Coefficients of MVP Model

In this subsection, we carry out the MVP model. Our model contains five independent equations, and the estimated correlation coefficients among the error terms of each equation are presented in Table 4. Most correlations between the error terms of the labor equation and chronic disease equations are statistically significant except the cases of labor force participation with diabetes in the presenile men group and with mental problems in the presenile women group. For example, the correlation coefficient between labor force participation and mental problems is significant at 0.58. Considering the significant correlations, a study on the relationship between chronic diseases and labor force participation is necessary for the aging problem of China. In terms of the correlation among the error terms of the four chronic diseases, cardiovascular diseases have a strong positive correlation with diabetes at 1% level, around 0.36 in all the four groups. Diabetes also has significant coefficients with mental problems, which demonstrates the existence of mutual interactions among different chronic diseases. The strong correlations among error terms indicate that the exogenous hypothesis of chronic diseases is not acceptable when studying their impact on labor force participation, and a separate analysis of labor force participation and chronic diseases is not applicable, that is why we choose the MVP model with simultaneous equations.

**Table 4:** Estimated correlation coefficients of a multivariate probit (MVP) model.

	**Presenile men**	**Presenile women**	**Senile men**	**Senile women**
*ρ_*lh*_*	0.1^**^	0.13^**^	0.35^***^	0.28^***^
*ρ_*ld*_*	0.09	0.24^**^	0.17^**^	0.25^***^
*ρ_*lm*_*	0.58^***^	0.09	0.53^***^	0.37^***^
*ρ_*lo*_*	0.25^***^	0.27^***^	0.19^***^	0.11^**^
*ρ_*hd*_*	0.37^***^	0.36^***^	0.35^***^	0.37^***^
*ρ_*hm*_*	−0.07	0.05	0.05	0.11^**^
*ρ_*ho*_*	0.02	0.11^*^	0.07^*^	0.04
*ρ_*dm*_*	0.11^***^	0.16^***^	0.08^***^	0.11^***^
*ρ_*do*_*	0.02	0.1^***^	0.03	0.06^**^
*ρ_*mo*_*	0.09^**^	0.14^***^	0.18^***^	0.07^**^

* *means significant at the 10% level;*

** *means significant at the 5% level;*

*** *means significant at the 1% level*.

The advantage of our MVP model is that we treate chronic diseases as endogenous, and we also consider the comorbidity of all chronic diseases by using five structural equations. In Table 5, we compare the MVP model with the bivariate probit (BVP) model, which does not consider the comorbidity and the univariate probit (UVP) model taking chronic diseases as exogenous. As presented in Table 5, the coefficients of the BVP model are much higher than the MVP model, that is, because of the existence of noise terms when ignoring the interaction of each chronic disease. When using five simultaneous equations and considering comorbidity, the coefficients turn smaller and reflect the real effect of chronic diseases. The coefficients of the UVP model are smaller than those of our MVP model, for example, the UVP coefficient of diabetes of the presenile women group is −0.44, which is smaller than the MVP coefficient of diabetes (−1.13). That is the case when chronic diseases are exogenous, however, the significant correlations in Tables 1, 4 indicate that taking chronic diseases as exogenous is unreasonable, thus our MVP model takes into account the endogeneity of chronic diseases.

**Table 5:** Estimated coefficients comparation of probit models.

	**Presenile Men**			**Presenile Women**		
	**MVP**	**BVP**	**UVP**	**MVP**	**BVP**	**UVP**
Card	−0.71^***^	−1.67^***^	−0.36^***^	−0.63^***^	−1.58^***^	−0.32^***^
Diab	−0.59^**^	−1.96^***^	−0.11^**^	−1.13^***^	−1.86^***^	−0.44^**^
Mental	−1.66^***^	−2.44^***^	−0.96^***^	−0.04	−2.22^***^	−0.44^***^
Othdis	−0.23^**^	−1.49^***^	−0.01	−0.06	−1.33^***^	−0.04
	**Senile Men**			**Senile Women**		
	**MVP**	**BVP**	**UVP**	**MVP**	**BVP**	**UVP**
Card	−0.85^***^	−1.64^***^	−0.50^***^	−0.61^***^	−1.64^***^	−0.44^***^
Diab	−0.40^**^	−1.71^***^	−0.32^**^	−0.39^**^	−2.03^***^	−0.33^***^
Mental	−1.08^***^	−2.35^***^	−0.18	−0.95^***^	−2.06^***^	−0.11
Othdis	−0.33^***^	−1.36^***^	−0.14^**^	−0.11	−1.33^***^	−0.12^***^

* *means significant at the 10% level;*

** *means significant at the 5% level;*

*** *means significant at the 1% level*.

Based on the abovementioned analysis, we have two conjectures: firstly, senile respondents have lower labor force participation than the presenile ones in both gender groups. Secondly, respondents with chronic diseases have lower labor force participation than the ones without chronic diseases. We check these conjectures in Table 6, where we present some conditional probabilities and treatment effects of each group. When considering only one chronic disease, consistent with our conjecture, both senile groups have lower labor force participation than presenile counterparts. For example, the labor force probability of presenile women conditioning on mental problems is 42.83%, which is lower than the probability without mental problems (52.43%). However, there are a few exceptions for cardiovascular diseases, the probabilities of labor force participation with cardiovascular diseases are higher compared to the probabilities without that kind of disease for both presenile groups and senile men. It also leads to the higher labor force participation probabilities with more than two chronic diseases including cardiovascular diseases. For example, for the presenile men group, the probability conditioning on cardiovascular diseases is 54.24%, much higher than 42.84%—the probability without cardiovascular diseases, meanwhile, the probability conditioning on cardiovascular diseases and mental problems is 52.94%, also higher than the probability without two kinds of chronic diseases (42.86%). One explanation is that for cardiovascular diseases, to pay for the diseases, respondents have to continue working. But for other diseases, the health conditions are too poor to continue working. This also causes the treatment effects of cardiovascular diseases to be quite different from other diseases, which are positive except for senile women.

**Table 6:** Predicted labor force participation probabilities (%).

	**Presenile women**	**Presenile men**	**Senile women**	**Senile men**
**Marginal probabilities**				
P (L=1)	52.14	53.17	41.19	43.64
**Conditional probabilities**				
P(L=1|Ch=0)	45.16	42.84	42.16	40.14
P(L=1|Ch=1)	53.75	54.24	40.28	45.89
P(L=1|Cd=0)	54.00	53.53	42.15	45.49
P(L=1|Cd=1)	46.55	52.02	39.89	40.75
P(L=1|Cm=0)	52.43	53.23	41.51	43.78
P(L=1|Cm=1)	42.83	51.40	36.56	41.22
P(L=1|Co=0)	52.37	53.23	41.32	43.83
P(L=1|Co=1)	38.73	42.72	35.00	25.53
**Treatment effects**				
P(L=1|Ch=1)–P(L=1|Ch=0)	8.59	11.41	−1.88	5.75
P(L=1|Cd=1)–P(L=1|Cd=0)	−7.45	−1.52	−2.27	−4.74
P(L=1|Cm=1)–P(L=1|Cm=0)	−9.60	−1.83	−4.95	−2.57
P(L=1|Co=1)–P(L=1|Co=0)	−13.64	−10.51	−6.31	−18.29
P(L=1|Cd=1,Cm=1)–P(L=1|Cd=0,Cm=0)	−15.96	−2.30	−6.04	−4.97
P(L=1|Ch=1,Cm=1)–P(L=1|Ch=0,Cm=0)	−0.36	10.08	−7.14	3.89
P(L=1|Ch=1,Cd=1,Cm=1)–P(L=1|Ch=0,Cd=0,Cm=0)	−6.81	9.86	−8.71	1.15
P(L=1|Ch=1,Cd=1,Cm=1,Co=1)–P(L=1|Ch=0,Cd=0,Cm=0,Co=0)	−15.38	4.10	−14.50	−13.05

As for diabetes, mental problems, and other diseases, the treatment effects are consistent. Among the four age-gender groups, presenile women are most vulnerable in face of chronic diseases when making working decisions, especially in the case of mental problems, the treatment effect of mental problems is −9.6, which is the highest among all the univariate treatment effects. As a comparison, the influence of chronic diseases on presenile men is the smallest among the four groups, indicating that presenile men face a higher burden of raising the family and cannot quit working even when they suffer from chronic diseases. As for the two senile groups, men are more influenced by physical conditions such as diabetes on their labor force participation decision, whereas women are easy to get affected by psychological problems. For instance, the treatment effect of diabetes for men is −4.74, higher than −2.27 of women and the treatment effect of mental problems for women is −4.95, higher than −2.57 of senile men. Furthermore, when suffering from more than one chronic disease, the labor force participation probability of women decreases dramatically compared with the case of only one chronic disease. Taking the presenile women as an example, compared to −7.45 of diabetes, the treatment effect increases to −15.96 when the respondent has both diabetes and mental problems. However, cases are different when it comes to men and cardiovascular diseases, there are two opposite effects of cardiovascular diseases on the labor force participation decision: on one hand, when suffering from cardiovascular diseases, men may reduce working intensity considering their poor-health conditions; on the other hand, as a Chinese tradition, men have a greater pressure of supporting the family, and the cost of cardiovascular diseases is in a long range, the expenses and family burden force men to continue working. We cannot define which side of the effects is more important, thus the treatment effect of cardiovascular diseases is uncertain.

### Marginal Effects of Exogenous Variables

In addition to the MVP coefficients and probabilities of conditioning on chronic diseases, we also calculate the marginal effects of all explanatory variables on the univariate probability of labor force participation. To analyze the exact impact of each exogenous variable, we divide it into two parts: direct marginal effects on the probability of labor force participation and indirect marginal effects through four chronic diseases. The socioeconomic variables, such as marital status and education conditions, have both direct and indirect effects on the probability of labor force participation, and the lifestyle variables, such as smoking, drinking habits, and exercise, only have indirect marginal effects *via* chronic diseases.

Tables 7A,B present the direct and indirect marginal effects for two presenile and senile groups, respectively. As for the effect of age and for all age-gender groups, the probability of labor force participation decreases with age. For example, taking men aged between 55 and 60 years as a reference, men aged between 50 and 54 years are 3.6% and more likely to join the labor force market, and men aged between 45 and 49 years are 4.9% and more likely to join the labor force market. The positive marginal effect of age mainly comes from the direct effect in the labor equation. The younger the age, the higher the probability of labor force participation.

**Table 7A:** Marginal effects on the labor force participation of presenile groups.

	**Direct**	**Indirect**				**Total**
		**Card**	**Diab**	**Mental**	**Othdis**	
**Male**						
AGE44	0.0824	−0.0107	0.0175	−0.0044	0.0001	0.085
AGE49	0.0411	0.0060	0.0005	−0.0009	0.0020	0.049^**^
AGE54	0.0362	−0.0003	−0.0004	0.0001	0.0004	0.036^**^
URBAN	−0.1042	−0.0098	−0.0017	−0.0008	0.0023	−0.114
MARRIED	0.0826	−0.0032	−0.0003	0.0052	0.0000	0.084
TOTWTH	0.0047	−0.0015	−0.0003	0.0009	0.0011	0.005^**^
NUMBER	−0.0009			0.0000		−0.001^**^
EDUHIG	−0.0160			0.0213		0.005
EDUMID	−0.0079			0.0003		−0.008^**^
MIDDRK		−0.0045	−0.0004	−0.0012	0.0010	−0.005^**^
HVYDRK		−0.0026	0.0001	−0.0011	0.0004	−0.003^**^
CURSMK		0.0048	0.0007	0.0012	−0.0021	0.005^**^
BEFSMK		−0.0046	−0.0005	−0.0003	−0.0018	−0.007^**^
EXH		0.0276	0.0032	0.0076	0.0101	0.049
EXM		0.0117	0.0015	0.0051	0.0038	0.022
EXL		0.0028	−0.0008	0.0051	0.0017	0.009
**Female**						
AGE44	0.0748	0.0188	0.0147	0.0001	0.0008	0.109^**^
AGE49	0.0786	0.0073	0.0029	0.0001	0.0005	0.089^**^
URBAN	−0.1708	−0.0066	−0.0051	−0.0001	0.0007	−0.182^**^
MARRIED	−0.0120	0.0143	−0.0035	0.0001	0.0004	−0.001^**^
TOTWTH	−0.0096	0.0026	0.0011	0.0001	0.0005	−0.005^**^
NUMBER	0.0063			0.0000		0.006^**^
EDUHIG	0.1245			0.0001		0.125^**^
EDUMID	−0.0447			0.0000		−0.045^**^
MIDDRK		−0.0144	0.0077	0.0000	−0.0007	−0.007^**^
HVYDRK		0.0119	0.0040	−0.0001	0.0005	0.016^**^
CURSMK		−0.0065	−0.0048	0.0003	0.0003	−0.011^**^
BEFSMK		−0.0057	−0.0007	−0.0001	−0.0039	−0.01^**^
EXH		0.0463	0.0164	0.0002	0.0064	0.069^**^
EXM		0.0258	0.0080	0.0001	0.0038	0.038^**^
EXL		0.0020	0.0044	0.0001	0.0000	0.007^**^

* *means significant at the 10% level;*

** *means significant at the 5% level;*

*** *means significant at the 1% level*.

**Table 7B:** Marginal effects on the labor force participation of senile groups.

	**Direct**	**Indirect**				**Total**
		**Card**	**Diab**	**Mental**	**Othdis**	
**Male**						
AGE64	0.4799	−0.0088	−0.0016	−0.0061	−0.0061	0.457^**^
AGE69	0.3790	−0.0117	−0.0014	−0.0058	−0.0123	0.348^**^
AGE74	0.2580	−0.0211	−0.0009	−0.0030	−0.0057	0.227^**^
URBAN	−0.2778	−0.0378	−0.0056	0.0004	0.0069	−0.314^**^
MARRIED	0.1490	−0.0067	−0.0025	−0.0002	−0.0061	0.133^**^
TOTWTH	−0.0225	−0.0069	−0.0006	0.0005	0.0005	−0.029^**^
NUMBER	−0.0017			0.0006		−0.001^**^
EDUHIG	−0.3166			0.0068		−0.31^**^
EDUMID	−0.1168			−0.0010		−0.118^**^
MIDDRK		0.0042	0.0011	0.0003	0.0039	0.01^**^
HVYDRK		0.0219	0.0035	0.0012	0.0024	0.029^**^
CURSMK		0.0218	0.0035	−0.0032	−0.0080	0.014^**^
BEFSMK		−0.0111	0.0003	−0.0066	−0.0132	−0.031^**^
EXH		0.0971	0.0053	0.0128	0.0341	0.149^**^
EXM		0.0530	0.0026	0.0100	0.0155	0.081^**^
EXL		0.0257	0.0048	0.0084	−0.0020	0.037^**^
**Female**						
AGE59	0.5681	0.0068	−0.0062	−0.0064	−0.0035	0.559^**^
AGE64	0.4905	−0.0032	−0.0101	−0.0069	−0.0050	0.465^**^
AGE69	0.4078	−0.0164	−0.0078	−0.0039	−0.0050	0.375^**^
AGE74	0.2283	−0.0121	−0.0088	−0.0044	−0.0030	0.200^**^
URBAN	−0.2752	−0.0266	−0.0074	0.0017	0.0022	−0.305^**^
MARRIED	0.1236	−0.0002	−0.0021	0.0013	−0.0006	0.122^**^
TOTWTH	−0.0326	−0.0027	−0.0005	0.0009	0.0003	−0.035^**^
NUMBER	0.0055			−0.0003		0.005^**^
EDUHIG	−0.2559			−0.0071		−0.263^*^
EDUMID	−0.2212			0.0017		−0.22^**^
MIDDRK		0.0453	0.0028	−0.0006	−0.0013	0.046^**^
HVYDRK		0.0081	0.0139	0.0016	−0.0019	0.022^**^
CURSMK		−0.0027	0.0046	−0.0070	−0.0017	−0.007^**^
BEFSMK		−0.0208	−0.0040	−0.0100	−0.0089	−0.044^**^
EXH		0.0701	0.0115	0.0109	0.0111	0.104^**^
EXM		0.0357	0.0067	0.0092	0.0046	0.056^**^
EXL		0.0247	0.0038	0.0064	−0.0007	0.034^**^

* *means significant at the 10% level;*

** *means significant at the 5% level;*

*** *means significant at the 1% level*.

Geographical location also has a significant effect on the probability of labor force participation. As can be seen for all groups, living in urban areas reduces the probability of labor force participation, and the effect is stronger for seniles. For instance, the probability of labor force participation decreases by 30.5% for senile women who live in urban areas and decreases by 18.2% for presenile women, taking living in the rural as a reference. That is reasonable under the background of Chinese national conditions, the urban areas have relatively a complete welfare and pension system, which will guarantee the basic life of aging population, but in rural areas, one needs to worry about livelihood even when he gets old and has to make money through work. Most of the indirect effects *via* chronic diseases are negative, indicating that in urban areas, people tend to reduce labor force participation when they suffer from chronic diseases. However, the case is different in rural areasas the welfare system is not sound, and aging people have to continue working even when they are suffering from chronic problems. That is also an urgent problem to be solved.

For a marginal effect of marriage conditions and for the two senile groups, the total effects are significantly positive and mainly come from direct effects in the labor equation, and the negative indirect effects *via* chronic equations offset a part of the positive direct effects. Compared with unmarried respondents, married men (women) have a 13.3% (12.2%) higher probability of labor force participation. As for the presenile groups, marriage has a different effect on labor force participation for men and women. Married men have a 8.4% high probability of participating in the labor force market, but the effect is not significant; married women have a 0.1% low probability of labor force participation, the direct effect is 1.2%, but mostly offset by an indirect effect from cardiovascular diseases. As the old saying goes “Men's work centers around outside, women's work centers around the home,” after getting married, women spend more time in taking care of the family and act as a housewife while men continue working to support the family.

As for income-related variables, we tried individual income, household total income, household per capita income, household wealth per capita, and the total household wealth (TOTWTH). The results show that the TOTWTH has a most significant impact on the labor force participation of aging Chinese. There is no doubt that senile respondents with higher household wealth have a low probability of labor force participation, the decrease ranges from 2.9% for men to 3.5% for women. For senile groups, there is a reasonable decline in the probability of labor force participation of women while the probability for men increases. However, the magnitude of both effects is smaller and only 0.5%, indicating that for preseniles, the impact of family wealth is not as important as the selection of other exogenous variables when making a labor force participation decision. Interestingly, the household number has a positive marginal effect for men and a negative marginal effect for women, a reasonable explanation is that women tend to join the labor force market to release the burden when the family number is large. The magnitude of household number is also as small as the total wealth, indicating that a marginal effect of number is not a main concern.

Regarding a marginal effect of education conditions, we divide the education conditions of respondents into three levels: low education (reference), mid-level education (EDUMID), and high education (EDUHIG). The data comes from the question “What is the highest level of education you have attained?” we define the education level as “low” if the answer of a respondent is lower than “elementary school,” as “mid-level” if the answer is “middle school, high school or vocational school,” as “high” if the answer is “college associate, bachelor, master, or doctoral degree.” As shown in the tables, compared with the low-level education, senile groups with middle/high-level education tend to have a low labor force participation, and the probability for men is high. For instance, the labor force participation dropped by 31% for high-educated men. That is the same for mid-educated preseniles, but the magnitude is much small, dropped only by 4.5% for women and 0.8% for men. The most different is high-educated presenile men. The higher the level of education, and the higher the probability of labor force participation. This is identical with the policy of reasonable utilization of talented human resources.

As for the marginal effect of lifestyles, we test the three kinds of lifestyles, which have a significant influence on the incidence of chronic diseases—drinking, smoking, and exercise habits. Lifestyles only show indirect effects through chronic diseases as they are not considered in the labor equation. For drinking habit, the alcohol in the CHARLS questionnaire include spirits, beer, and wine, and we clarify drinking levels using the international standard drink. Based on the questionnaire, we define the drinking level as MIDDRK if the weekly standard drink is <7 or the daily standard drink is <3; as HVYDRK if the weekly standard drink is more than seven or the daily standard drink more than three. In Table 7B, compared with no drinking, moderate, and HVYDRK, both increase the probability of the labor force participation of seniles, which range from 1 to 4.6%, the effects mainly come from indirect effects *via* physical conditions. As for the presenile groups in Table 7A, the marginal effects of moderate and HVYDRK for men are negative. The results are consistent with Balsa and French (22) who proposed that intemperance is a thorny problem for presenile men, which can lead to a host of social issues. As for women, MIDDRK will reduce their probability of labor force participation by 0.7%, whereas HVYDRK will increase the probability by 1.6%, they need working to get money for their bad habits and the consequent expenses for chronic diseases (23).

As for smoking habits, our data comes from the two questions “Have you ever chewed tobacco, smoked a pipe, smoked self-rolled cigarettes, or smoked cigarettes/cigars” and “Do you still have the habit or have you totally quit.” According to the answers, we clarify smoking to the three levels: no smoking, BEFSMK, and CURSMK. For women, both smoking habits have negative marginal effects on the probability of labor force participation and the effects range from 0.7 to 4.4%, the mechanism is that bad smoking habits lead to the invasion of chronic diseases, which indirectly affect the labor force participation. For men, CURSMK and BEFSMK have the opposite effect. Smoking history has a negative marginal effect on the probability for both presenile and senile men; CURSMK has a positive effect on labor force participation, one explanation is that smoking can be seen as a way to relieve pressure as men may feel stressful in raising the family (24).

For the marginal effects of exercise, we clarify exercise to the three levels based on exercise hours and the question “Do you have any difficulty running/walking about…km”—low-level exercise (EXL), mid-level exercise (EXM), and high-level exercise (EXH) and taking no exercise as a reference. As shown in the table, all exercise levels have a positive effect on the probability of labor force participation, but the effect for presenile men is not significant. The higher the exercise level, the higher the increase of labor force participation probability. Taking presenile men as an example, respondents with EXL have a 0.7% high probability to join the labor force market, the increase improves to 3.8% for the middle-level exercise respondents, and 6.9% for the EXH respondents. Furthermore, the positive exercise effect is more beneficial to senile groups, especially senile men.

## Discussion and Conclusion

Our article aims to study the impact of chronic diseases on the labor force participation among presenile and senile Chinese. By comparing the different models, we find that the MVP model is the most effective one when considering the significant correlations and the endogeneity of chronic diseases (25). We use a system equation method and estimate the five equations, including the labor force participation and four kinds of chronic diseases. Most of our estimated correlations are statistically significant. Our sample is derived from the CHARLS database, and the empirical results include various probabilities, treatment effects, and the marginal effects of exogenous variables on the probability of labor force participation separated by age and gender (presenile men, presenile women, senile men, and senile women). The prevalence of chronic diseases especially among the aging population has profound a negative impact on the labor force participation in China. This article helps to identify the risk factors for labor force participation and to measure the impact level of chronic diseases on labor force participation.

The analysis reveals some useful results. Firstly, in general, when suffering from epidemic chronic diseases, the labor force participation of the population aged more than 45 years shows a downward trend, the impact of chronic diseases on labor force participation is most obvious for presenile women with the treatment effect as high as 15.96 under the conditions of diabetes and mental problems. In addition, living under high pressure nowadays, mental problems have become a prevailing chronic disease affecting the aging Chinese. As can be seen from the MVP-estimated coefficients, the impact of mental problems is much severe than the physical problems especially for men, psychological health consulting and services are in urgent need. Generally speaking, the prevention of chronic diseases is necessary, the government needs to make their people aware of the adverse effects of chronic diseases both on themselves and on the social economy.

Sociodemographic exogenous variables also have direct and indirect effects on the probability of labor force participation. The age bonus of labor force participation disappears as one gets elder. The population in urban areas enjoys more social welfare while the rural elderly is suffering, the developing strategy of Chinese urbanization needs to be adjusted and tilted to rural areas and aging people. Family wealth and family number are not the main concerns when making a labor force participation decision for Chinese aged more than 45 years. Regarding the lifestyles that have direct effects on chronic diseases and indirect effects on labor force participation, we also get some intuitive conclusions—a good way of maintaining a healthy lifestyle for aging people to be immune to chronic diseases and stay in the labor force market.

This article selects the sample of China—a developing country to study the relationship between the labor force participation and four kinds of chronic diseases. It is an important supplement to current literature studies on labor force participation as most studies of this area focus on advanced economies such as Australia. Based on our empirical results, we also give some practical suggestions, which are helpful for the aging problem of China.

## Data Availability Statement

The datasets presented in this study can be found in online repositories. The names of the repository/repositories and accession number(s) can be found below: http://charls.pku.edu.cn/.

## Author Contributions

XQ analyzed questionaire, carried out the experiments, interpreted the results, and prepared the manuscript. BW supervised and reviewed the final version. HG edited the final paper and is in charge of project administration and funding acquisition. All authors read and approved the final manuscript.

## Funding

This study was supported by the National Key Research and Development Program of China (2020YFB1006104), the National Natural Science Foundation of China (71773025), and the Fundamental Research Funds for the Central Universities (Grant No. HIT.HSS.202115).

## Conflict of Interest

The authors declare that the research was conducted in the absence of any commercial or financial relationships that could be construed as a potential conflict of interest.

## Publisher's Note

All claims expressed in this article are solely those of the authors and do not necessarily represent those of their affiliated organizations, or those of the publisher, the editors and the reviewers. Any product that may be evaluated in this article, or claim that may be made by its manufacturer, is not guaranteed or endorsed by the publisher.

## Figures and Tables

**Table:** 

**Acronym**	**Definition**
L/Labor	1 if in labor force, 0 otherwise
Ch/card	1 if having cardiovascular diseases now, 0 otherwise
Cd/diab	1 if having diabetes now, 0 otherwise
Cm/mental	1 if having mental problems, 0 other wise
Co/othdis	1 if having other chronic diseases, 0 otherwise
AGE44	1 if age younger than 45, 0 otherwise
AGE49	1 if age between 45 and 49, 0 otherwise
AGE54	1 if age between 50 and 54, 0 otherwise
AGE59	1 if age between 55 and 60, 0 otherwise(referenced group)
URBAN	1 if in urban area, 0 if in rural area
MARRIED	1 if married, 0 otherwise
TOTWTH	Total wealth of household
NUMBER	Fanily numbers of household
EDUHIG	1 if had high level education, 0 otherwise
EDUMID	1 if had middle level education, 0 otherwise
CURSMK	1 if currently smoking, 0 otherwise
BEFSMK	1 if before smoking, 0 otherwise
MIDDRK	1 if male ≤ 14 per week(4 per day), female ≤ 7 per week(3 per day), 0 otherwise
HVYDRK	1 if heavy level drink, 0 otherwise
EXH	1 if high level exercise, 0 otherwise
EXM	1 if middle level exercise, 0 otherwise
EXL	1 if low level exercise, 0 otherwise
